# Supplementary material for: Psiadin and plectranthone selectively inhibit colorectal carcinoma cells proliferation via modulating cyclins signaling and apoptotic pathways
Source: PLoS One. 2021 Jun 4;16(6):e0252820. doi: 10.1371/journal.pone.0252820 (PMC8177666; doi:10.1371/journal.pone.0252820)
Supplement: S1 Table — (PDF) [file pone.0252820.s001.pdf]

**S1 Table. Antibodies, their target proteins and sources**

| Target protein | Antibody | Cat # | Dilution | Source |
|---|---|---|---|---|
| BCLX | BCLX (54H6) Rabbit mAb | 2764S | 1:1000 | Cell Signaling Technology |
| MCL-1 | MCL-1 (D2W9E) Rabbit mAb | 94296S | | |
| Cyclin A2 | Cyclin A2 (E1D9T) Rabbit mAb | 91500S | | |
| Cyclin E1 | Cyclin E1 (D7T3U) Rabbit mAb | 20808S | | |
| Cyclin B1 | Cyclin B1 (D5C10) XP® Rabbit mAb | 12231S | | |
| Cyclin D1 | Cyclin D1 (92G2) Rabbit mAb | 2978S | | |
| CDK2 | CDK2 (78B2) Rabbit mAb | 2546S | | |
| CDK4 | CDK4 (D9G3E) Rabbit mAb | 12790S | | |
| CDK6 | CDK6 (D4S8S) Rabbit mAb | 13331S | | |
| β-actin | β-actin (13E5) Rabbit mAb | 4970S | | |
| AIF | AIF (D39D2) XP® Rabbit mAb | 5318S | | |
| BID | BID Rabbit Antibody (Human Specific) | 2002S | | |
| BAK | BAK (D4E4) Rabbit mAb | 12105S | | |
| Cleaved Caspase-3 | Cleaved Caspase-3 (Asp175) (5A1E) Rabbit mAb | 9664S | | |
| Caspase-8 | Caspase-8 (D35G2) Rabbit mAb #4790 | 4790S | | |
| Cleaved Caspase-8 | Cleaved Caspase-8 (Asp391) (18C8) Rabbit mAb | 9496S | | |
| Cleaved Caspase-9 | Cleaved Caspase-9 (Asp315) (D8I9E) Rabbit mAb | 20750S | | |
| CIAP2 | CIAP2 (58C7) Rabbit mAb | 3130S | | |
| CIAP1 | CIAP1 (D5G9) Rabbit mAb | 7065S | | |
| FLIP | FLIP (D16A8) Rabbit mAb | 8510S | | |
| AKT | AKT (pan) (C67E7) Rabbit mAb | 4691S | | |
| pAKT | Phospho-AKT (Ser473) (193H12) Rabbit mAb | 4058S | | |
| c-RAF | c-RAF (D4B3J) Rabbit mAb | 53745S | | |
| MEK1/2 | MEK1/2 (D1A5) Rabbit mAb | 8727S | | |
| pMEK1/2 | Phospho-MEK1/2 (Ser221) (166F8) Rabbit mAb | 2338S | | |
| p38 MAPK | p38 MAPK (D13E1) XP® Rabbit mAb | 8690S | | |
| PARP | PARP (46D11) Rabbit mAb | 9532S | | |
| SMAC/Diablo | SMAC/Diablo (D5S3R) Rabbit mAb | 15108S | | |
| Cyto c | Cytochrome c (D18C7) Rabbit mAb | 11940S | | |
| RB | RB (D20) Rabbit mAb | 9313S | | |
| pRB | Phospho-RB (Ser807/811) (D20B12) XP® Rabbit mAb | 8516S | | |
