# Supplementary material for: Psiadin and plectranthone selectively inhibit colorectal carcinoma cells proliferation via modulating cyclins signaling and apoptotic pathways
Source: PLoS One. 2021 Jun 4;16(6):e0252820. doi: 10.1371/journal.pone.0252820 (PMC8177666; doi:10.1371/journal.pone.0252820)

S1 Raw image. Protein expression in CCL235 cells. Original X-ray films of the western blot membrane strips are shown here, of the corresponding cropped images shown in Fig 5. Lanes 1 and 2 from the left are the relevant lanes that correspond to Veh and Ps-treated samples. Lanes marked with X are not included in Fig 5 and are not relevant to this study.

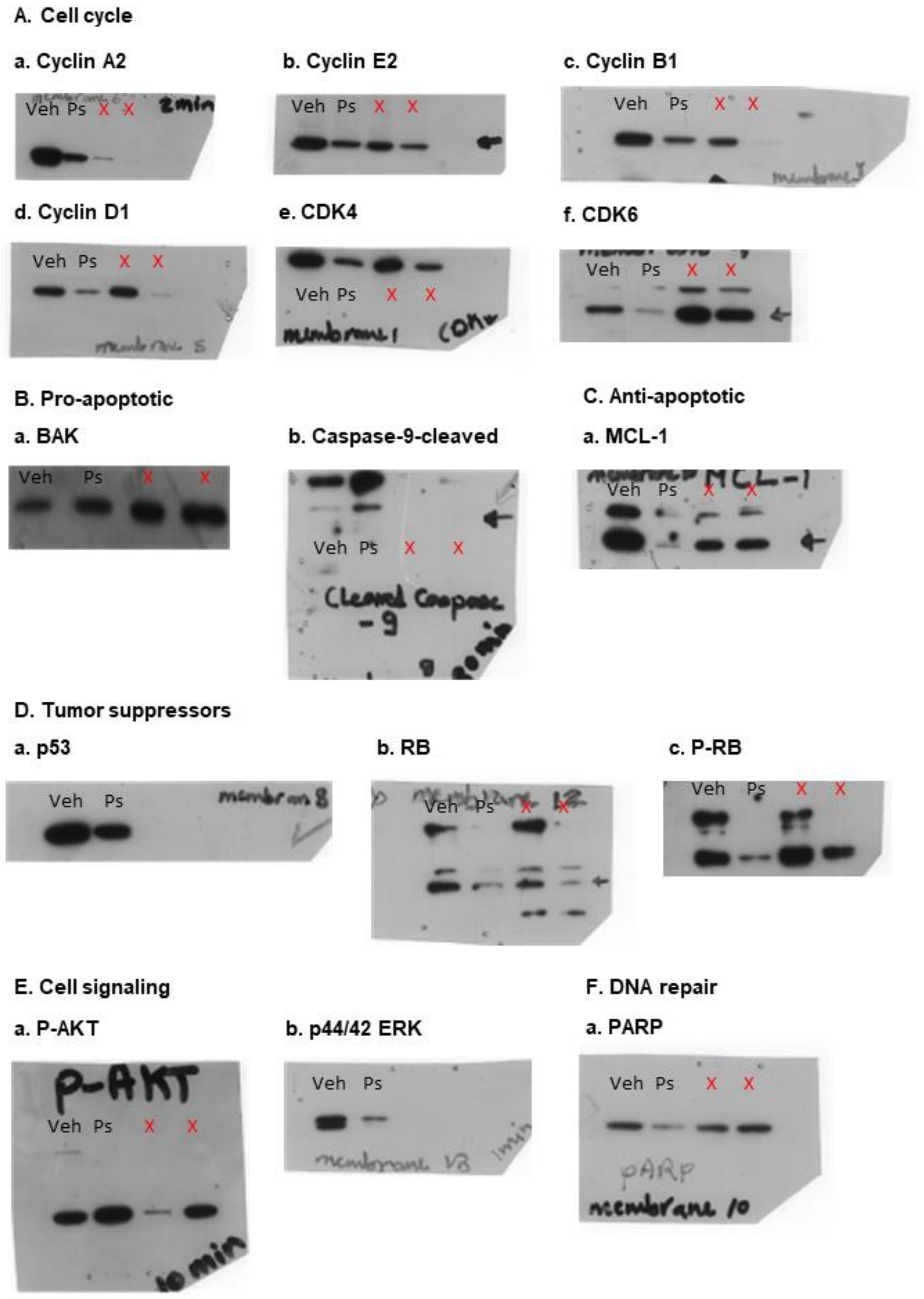

Supplement: S1 Raw image — Original X-ray films of the western blot membrane strips are shown here, of the corresponding cropped images shown in Fig 5. Lanes 1 and 2 from the left are the relevant lanes that correspond to Veh and Ps-treated samples. Lanes marked with X are not included in Fig 5 and are not relevant to this study. (PDF) [file pone.0252820.s002.pdf]
