# Supplementary material for: Psiadin and plectranthone selectively inhibit colorectal carcinoma cells proliferation via modulating cyclins signaling and apoptotic pathways
Source: PLoS One. 2021 Jun 4;16(6):e0252820. doi: 10.1371/journal.pone.0252820 (PMC8177666; doi:10.1371/journal.pone.0252820)

S2 Raw image. Actin expression in CCL235 cells. Original X-ray films of the western blot membrane strips are shown here of the actin bands (loading control) corresponding to each of the protein targets as indicated, which are shown as cropped images in Fig 5. Lanes 1 and 2 from the left are the relevant lanes that correspond to Veh and Ps-treated samples. Lanes or bands marked X are not included in Fig 5 and are not relevant to this study.

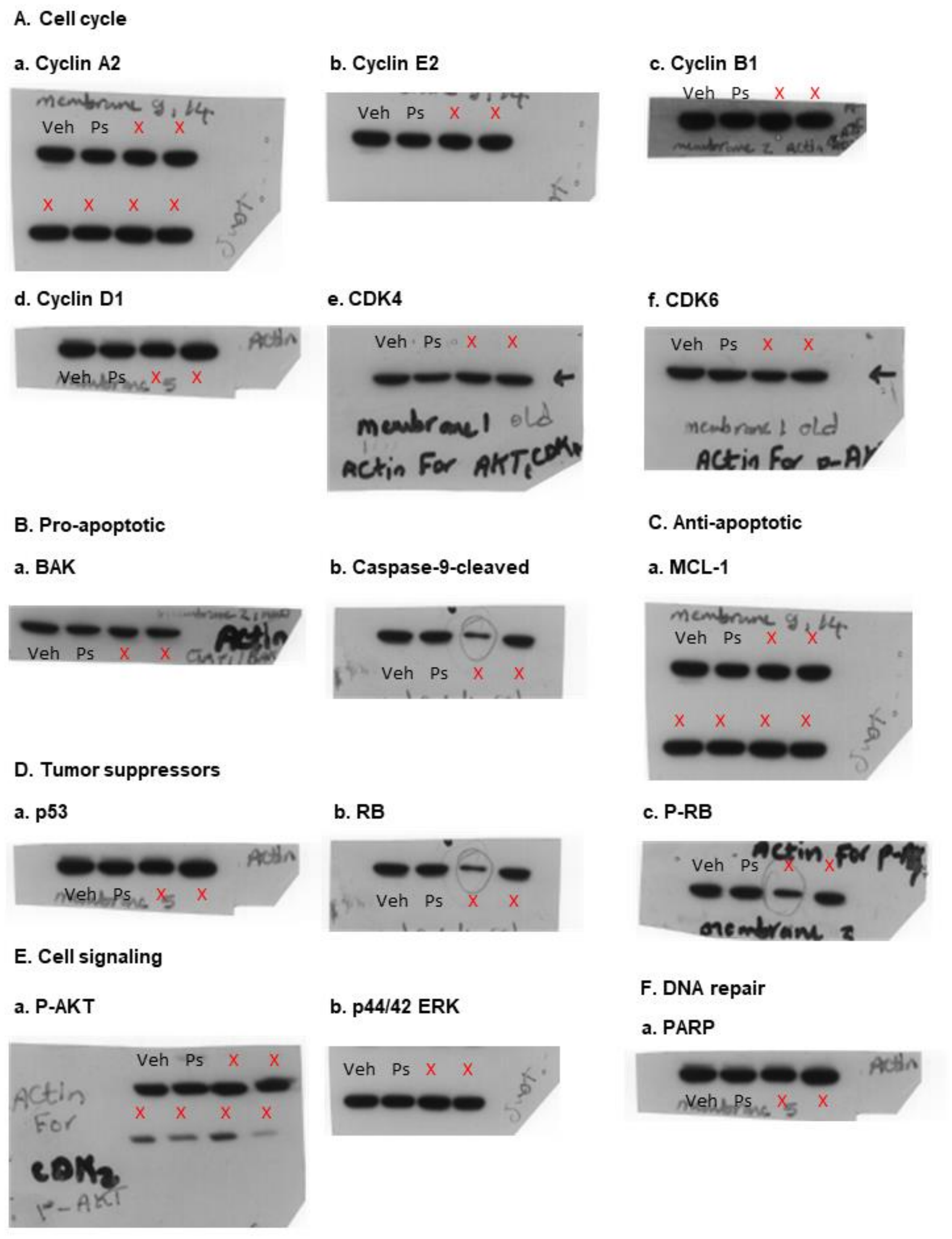

Supplement: S2 Raw image — Original X-ray films of the western blot membrane strips are shown here of the actin bands (loading control) corresponding to each of the protein targets as indicated, which are shown as cropped images in Fig 5. Lanes 1 and 2 from the left are the relevant lanes that correspond to Veh and Ps-treated samples. Lanes or bands marked X are not included in Fig 5 and are not relevant to this study. (PDF) [file pone.0252820.s003.pdf]
